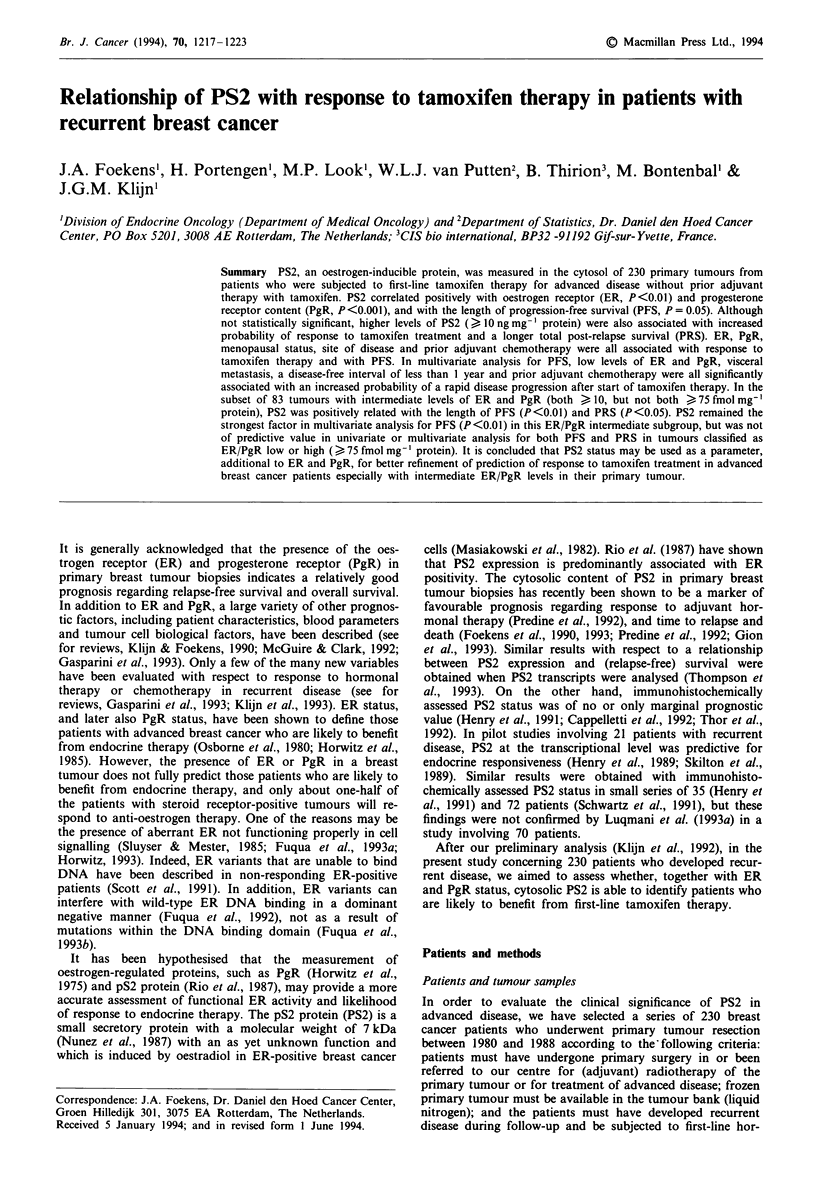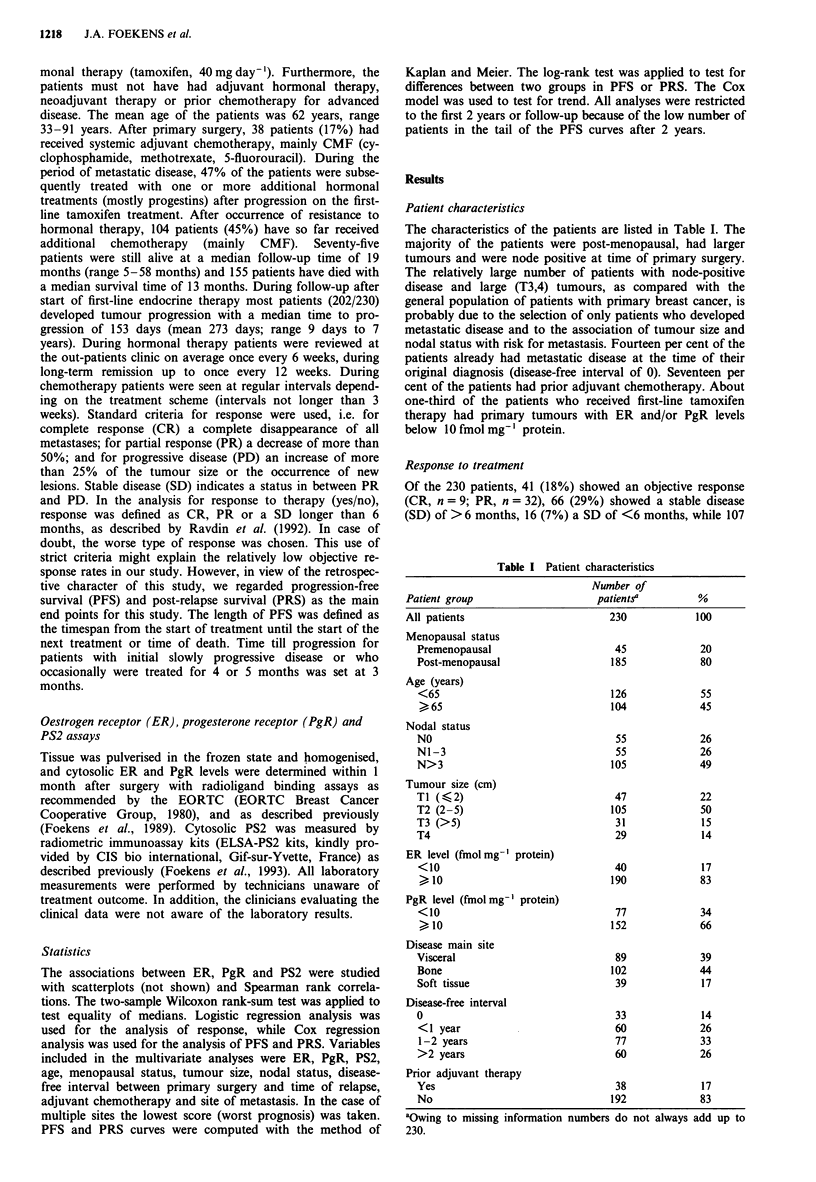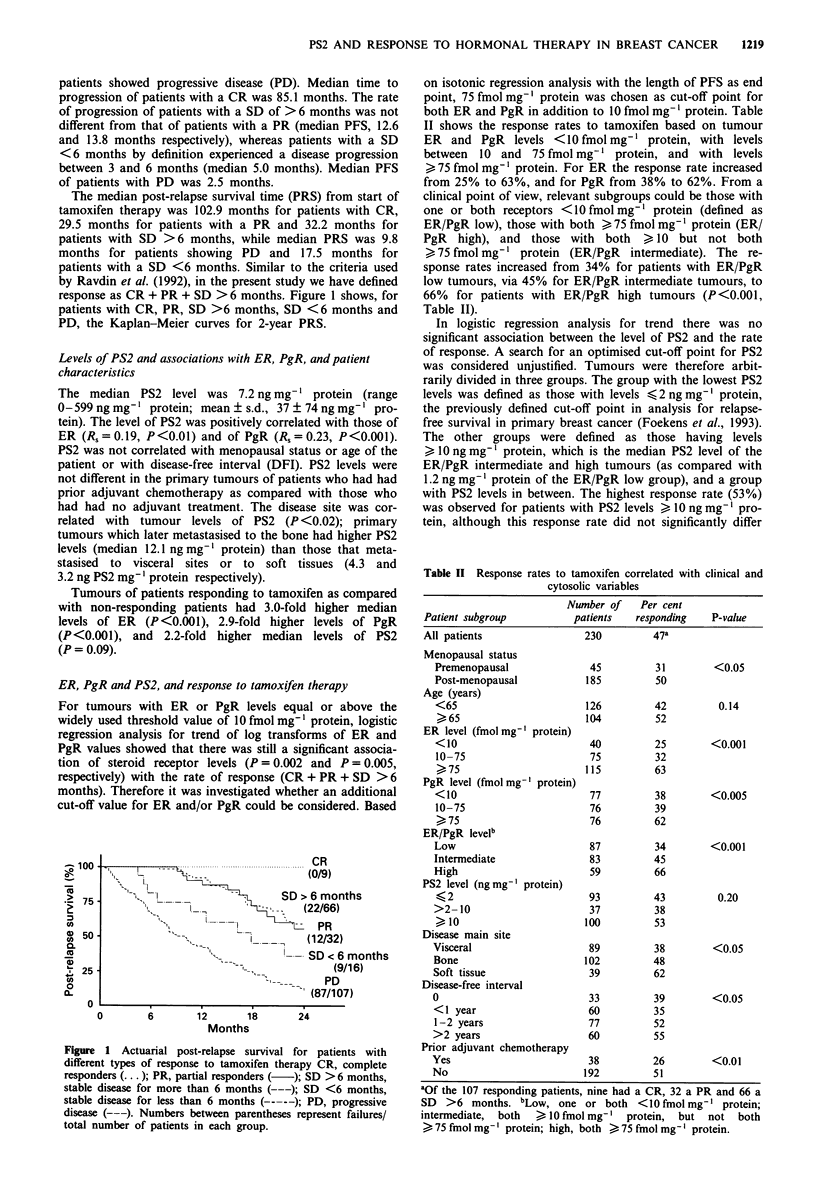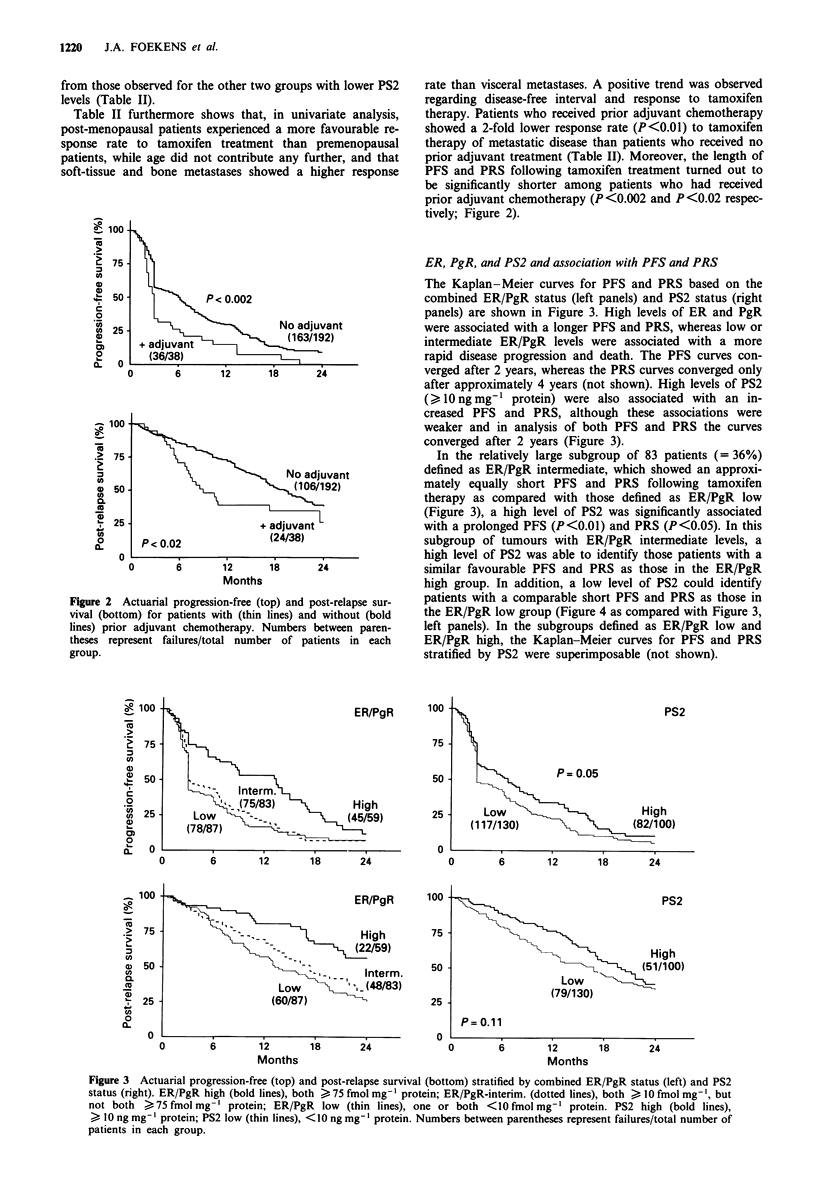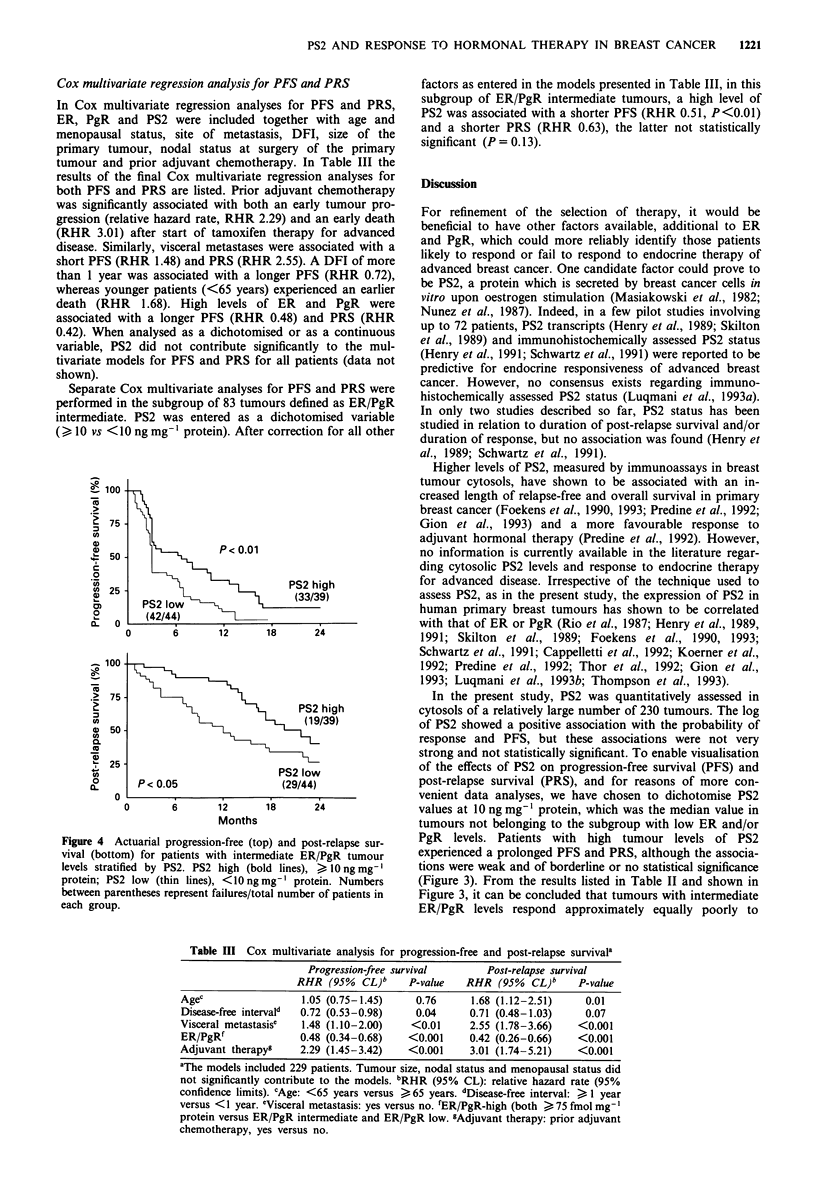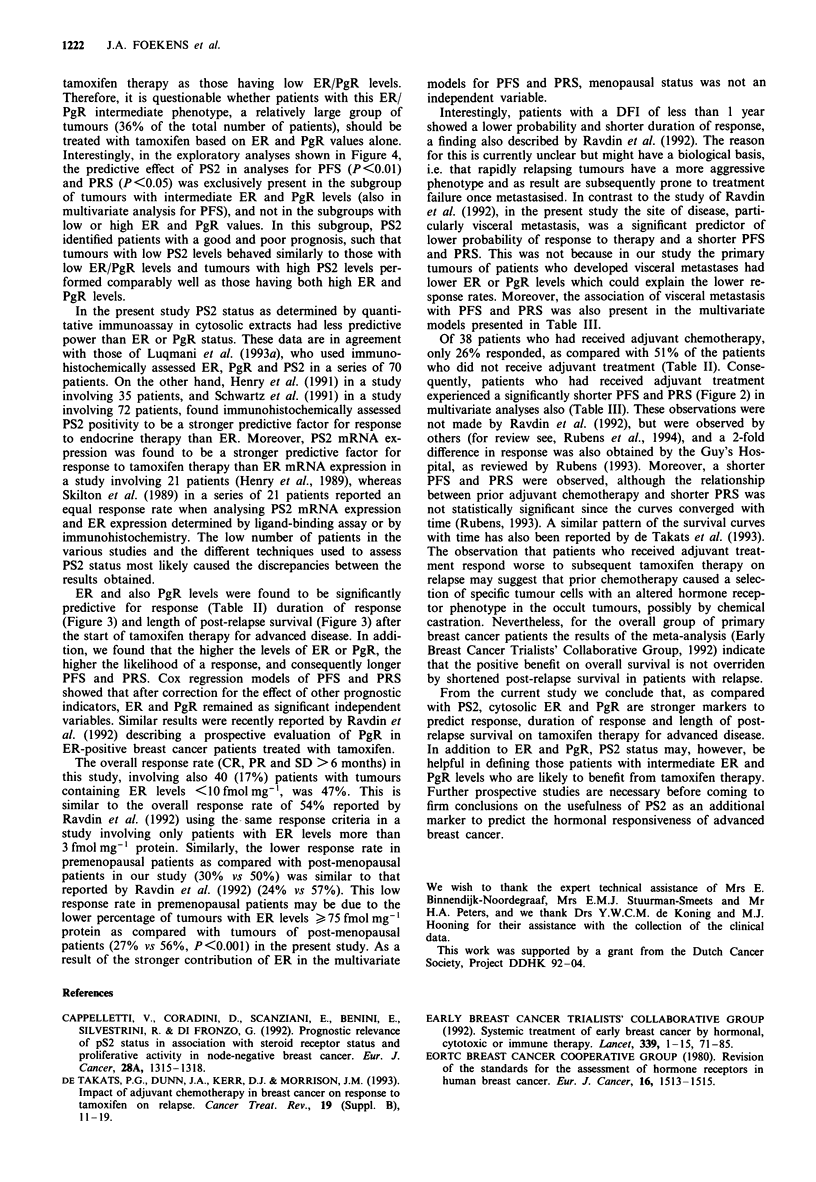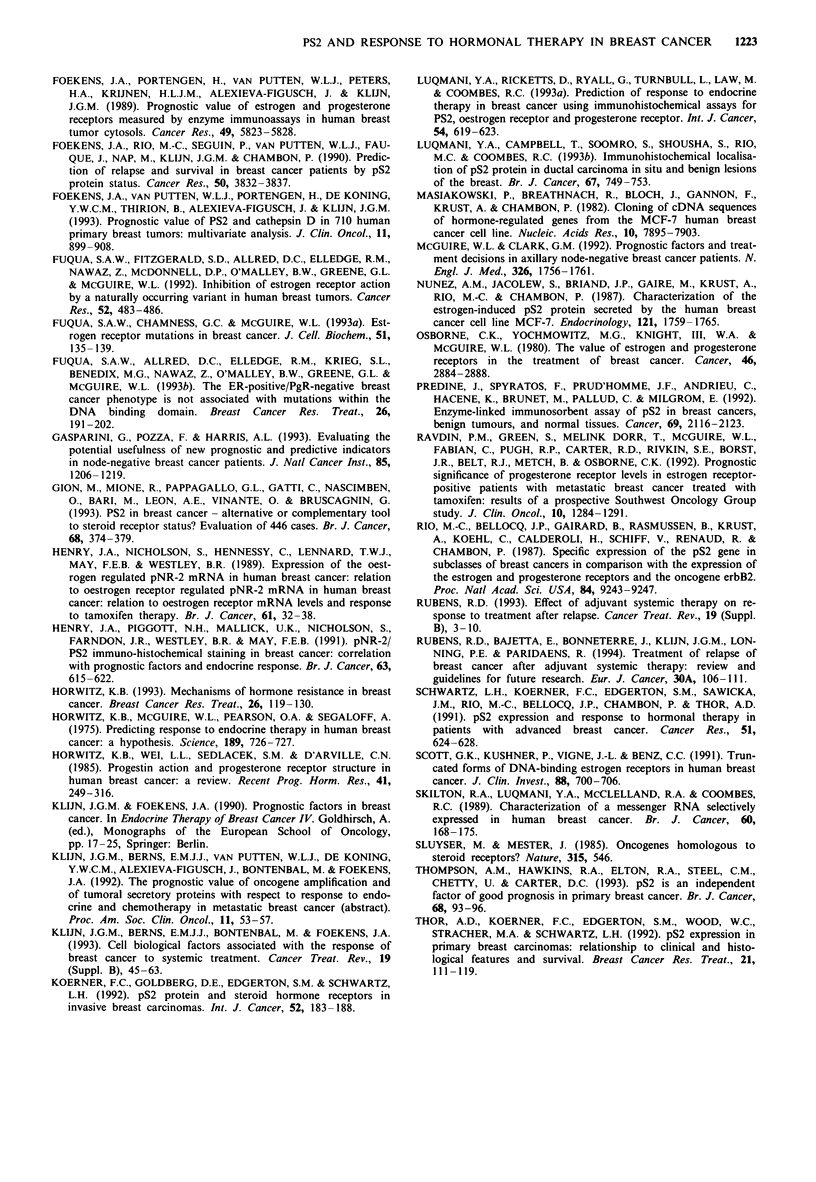# Relationship of PS2 with response to tamoxifen therapy in patients with recurrent breast cancer.

**DOI:** 10.1038/bjc.1994.476

**Published:** 1994-12

**Authors:** J. A. Foekens, H. Portengen, M. P. Look, W. L. van Putten, B. Thirion, M. Bontenbal, J. G. Klijn

**Affiliations:** Division of Endocrine Oncology (Department of Medical Oncology), Dr. Daniel den Hoed Cancer Center, Rotterdam, The Netherlands.

## Abstract

PS2, an oestrogen-inducible protein, was measured in the cytosol of 230 primary tumours from patients who were subjected to first-line tamoxifen therapy for advanced disease without prior adjuvant therapy with tamoxifen. PS2 correlated positively with oestrogen receptor (ER, P < 0.01) and progesterone receptor content (PgR, P < 0.001), and with the length of progression-free survival (PFS, P = 0.05). Although not statistically significant, higher levels of PS2 (> or = 10 ng mg-1 protein) were also associated with increased probability of response to tamoxifen treatment and a longer total post-relapse survival (PRS). ER, PgR, menopausal status, site of disease and prior adjuvant chemotherapy were all associated with response to tamoxifen therapy and with PFS. In multivariate analysis for PFS, low levels of ER and PgR, visceral metastasis, a disease-free interval of less than 1 year and prior adjuvant chemotherapy were all significantly associated with an increased probability of a rapid disease progression after start of tamoxifen therapy. In the subset of 83 tumours with intermediate levels of ER and PgR (both > or = 10, but not both > or = 75 fmol mg-1 protein), PS2 was positively related with the length of PFS (P < 0.01) and PRS (P < 0.05). PS2 remained the strongest factor in multivariate analysis for PFS (P < 0.01) in this ER/PgR intermediate subgroup, but was not of predictive value in univariate or multivariate analysis for both PFS and PRS in tumours classified as ER/PgR low or high (> or = 75 fmol mg-1 protein). It is concluded that PS2 status may be used as a parameter, additional to ER and PgR, for better refinement of prediction of response to tamoxifen treatment in advanced breast cancer patients especially with intermediate ER/PgR levels in their primary tumour.


Br. J. Cancer (1994), 70, 1217–1223 © Macmillan Press Ltd., 1994

### Relationship of PS2 with response to tamoxifen therapy in patients with recurrent breast cancer

J.A. Foekens[1], H. Portengen[1], M.P. Look[1], W.L.J. van Putten[2], B. Thirion[3], M. Bontenbal[1] & J.G.M. Klijn[1]

[1]*Division of Endocrine Oncology (Department of Medical Oncology) and* [2]*Department of Statistics, Dr. Daniel den Hoed Cancer Center, PO Box 5201, 3008 AE Rotterdam, The Netherlands;* [3]*CIS bio international, BP32 -91192 Gif-sur-Yvette, France.*

**Summary** PS2, an oestrogen-inducible protein, was measured in the cytosol of 230 primary tumours from patients who were subjected to first-line tamoxifen therapy for advanced disease without prior adjuvant therapy with tamoxifen. PS2 correlated positively with oestrogen receptor (ER, $P<0.01$) and progesterone receptor content (PgR, $P<0.001$), and with the length of progression-free survival (PFS, $P = 0.05$). Although not statistically significant, higher levels of PS2 ($\geqslant 10$ ng mg$^{-1}$ protein) were also associated with increased probability of response to tamoxifen treatment and a longer total post-relapse survival (PRS). ER, PgR, menopausal status, site of disease and prior adjuvant chemotherapy were all associated with response to tamoxifen therapy and with PFS. In multivariate analysis for PFS, low levels of ER and PgR, visceral metastasis, a disease-free interval of less than 1 year and prior adjuvant chemotherapy were all significantly associated with an increased probability of a rapid disease progression after start of tamoxifen therapy. In the subset of 83 tumours with intermediate levels of ER and PgR (both $\geqslant 10$, but not both $\geqslant 75$ fmol mg$^{-1}$ protein), PS2 was positively related with the length of PFS ($P<0.01$) and PRS ($P<0.05$). PS2 remained the strongest factor in multivariate analysis for PFS ($P<0.01$) in this ER/PgR intermediate subgroup, but was not of predictive value in univariate or multivariate analysis for both PFS and PRS in tumours classified as ER/PgR low or high ($\geqslant 75$ fmol mg$^{-1}$ protein). It is concluded that PS2 status may be used as a parameter, additional to ER and PgR, for better refinement of prediction of response to tamoxifen treatment in advanced breast cancer patients especially with intermediate ER/PgR levels in their primary tumour.

It is generally acknowledged that the presence of the oestrogen receptor (ER) and progesterone receptor (PgR) in primary breast tumour biopsies indicates a relatively good prognosis regarding relapse-free survival and overall survival. In addition to ER and PgR, a large variety of other prognostic factors, including patient characteristics, blood parameters and tumour cell biological factors, have been described (see for reviews, Klijn & Foekens, 1990; McGuire & Clark, 1992; Gasparini *et al.*, 1993). Only a few of the many new variables have been evaluated with respect to response to hormonal therapy or chemotherapy in recurrent disease (see for reviews, Gasparini *et al.*, 1993; Klijn *et al.*, 1993). ER status, and later also PgR status, have been shown to define those patients with advanced breast cancer who are likely to benefit from endocrine therapy (Osborne *et al.*, 1980; Horwitz *et al.*, 1985). However, the presence of ER or PgR in a breast tumour does not fully predict those patients who are likely to benefit from endocrine therapy, and only about one-half of the patients with steroid receptor-positive tumours will respond to anti-oestrogen therapy. One of the reasons may be the presence of aberrant ER not functioning properly in cell signalling (Sluyser & Mester, 1985; Fuqua *et al.*, 1993*a*; Horwitz, 1993). Indeed, ER variants that are unable to bind DNA have been described in non-responding ER-positive patients (Scott *et al.*, 1991). In addition, ER variants can interfere with wild-type ER DNA binding in a dominant negative manner (Fuqua *et al.*, 1992), not as a result of mutations within the DNA binding domain (Fuqua *et al.*, 1993*b*).

It has been hypothesised that the measurement of oestrogen-regulated proteins, such as PgR (Horwitz *et al.*, 1975) and pS2 protein (Rio *et al.*, 1987), may provide a more accurate assessment of functional ER activity and likelihood of response to endocrine therapy. The pS2 protein (PS2) is a small secretory protein with a molecular weight of 7 kDa (Nunez *et al.*, 1987) with an as yet unknown function and which is induced by oestradiol in ER-positive breast cancer cells (Masiakowski *et al.*, 1982). Rio *et al.* (1987) have shown that PS2 expression is predominantly associated with ER positivity. The cytosolic content of PS2 in primary breast tumour biopsies has recently been shown to be a marker of favourable prognosis regarding response to adjuvant hormonal therapy (Predine *et al.*, 1992), and time to relapse and death (Foekens *et al.*, 1990, 1993; Predine *et al.*, 1992; Gion *et al.*, 1993). Similar results with respect to a relationship between PS2 expression and (relapse-free) survival were obtained when PS2 transcripts were analysed (Thompson *et al.*, 1993). On the other hand, immunohistochemically assessed PS2 status was of no or only marginal prognostic value (Henry *et al.*, 1991; Cappelletti *et al.*, 1992; Thor *et al.*, 1992). In pilot studies involving 21 patients with recurrent disease, PS2 at the transcriptional level was predictive for endocrine responsiveness (Henry *et al.*, 1989; Skilton *et al.*, 1989). Similar results were obtained with immunohistochemically assessed PS2 status in small series of 35 (Henry *et al.*, 1991) and 72 patients (Schwartz *et al.*, 1991), but these findings were not confirmed by Luqmani *et al.* (1993*a*) in a study involving 70 patients.

After our preliminary analysis (Klijn *et al.*, 1992), in the present study concerning 230 patients who developed recurrent disease, we aimed to assess whether, together with ER and PgR status, cytosolic PS2 is able to identify patients who are likely to benefit from first-line tamoxifen therapy.

### Patients and methods

### *Patients and tumour samples*

In order to evaluate the clinical significance of PS2 in advanced disease, we have selected a series of 230 breast cancer patients who underwent primary tumour resection between 1980 and 1988 according to the following criteria: patients must have undergone primary surgery in or been referred to our centre for (adjuvant) radiotherapy of the primary tumour or for treatment of advanced disease; frozen primary tumour must be available in the tumour bank (liquid nitrogen); and the patients must have developed recurrent disease during follow-up and be subjected to first-line hor-

Correspondence: J.A. Foekens, Dr. Daniel den Hoed Cancer Center, Groen Hilledijk 301, 3075 EA Rotterdam, The Netherlands.
Received 5 January 1994; and in revised form 1 June 1994.

monal therapy (tamoxifen, 40 mg day$^{-1}$). Furthermore, the patients must not have had adjuvant hormonal therapy, neoadjuvant therapy or prior chemotherapy for advanced disease. The mean age of the patients was 62 years, range 33–91 years. After primary surgery, 38 patients (17%) had received systemic adjuvant chemotherapy, mainly CMF (cyclophosphamide, methotrexate, 5-fluorouracil). During the period of metastatic disease, 47% of the patients were subsequently treated with one or more additional hormonal treatments (mostly progestins) after progression on the first-line tamoxifen treatment. After occurrence of resistance to hormonal therapy, 104 patients (45%) have so far received additional chemotherapy (mainly CMF). Seventy-five patients were still alive at a median follow-up time of 19 months (range 5–58 months) and 155 patients have died with a median survival time of 13 months. During follow-up after start of first-line endocrine therapy most patients (202/230) developed tumour progression with a median time to progression of 153 days (mean 273 days; range 9 days to 7 years). During hormonal therapy patients were reviewed at the out-patients clinic on average once every 6 weeks, during long-term remission up to once every 12 weeks. During chemotherapy patients were seen at regular intervals depending on the treatment scheme (intervals not longer than 3 weeks). Standard criteria for response were used, i.e. for complete response (CR) a complete disappearance of all metastases; for partial response (PR) a decrease of more than 50%; and for progressive disease (PD) an increase of more than 25% of the tumour size or the occurrence of new lesions. Stable disease (SD) indicates a status in between PR and PD. In the analysis for response to therapy (yes/no), response was defined as CR, PR or a SD longer than 6 months, as described by Ravdin *et al.* (1992). In case of doubt, the worse type of response was chosen. This use of strict criteria might explain the relatively low objective response rates in our study. However, in view of the retrospective character of this study, we regarded progression-free survival (PFS) and post-relapse survival (PRS) as the main end points for this study. The length of PFS was defined as the timespan from the start of treatment until the start of the next treatment or time of death. Time till progression for patients with initial slowly progressive disease or who occasionally were treated for 4 or 5 months was set at 3 months.

### *Oestrogen receptor (ER), progesterone receptor (PgR) and PS2 assays*

Tissue was pulverised in the frozen state and homogenised, and cytosolic ER and PgR levels were determined within 1 month after surgery with radioligand binding assays as recommended by the EORTC (EORTC Breast Cancer Cooperative Group, 1980), and as described previously (Foekens *et al.*, 1989). Cytosolic PS2 was measured by radiometric immunoassay kits (ELSA-PS2 kits, kindly provided by CIS bio international, Gif-sur-Yvette, France) as described previously (Foekens *et al.*, 1993). All laboratory measurements were performed by technicians unaware of treatment outcome. In addition, the clinicians evaluating the clinical data were not aware of the laboratory results.

### *Statistics*

The associations between ER, PgR and PS2 were studied with scatterplots (not shown) and Spearman rank correlations. The two-sample Wilcoxon rank-sum test was applied to test equality of medians. Logistic regression analysis was used for the analysis of response, while Cox regression analysis was used for the analysis of PFS and PRS. Variables included in the multivariate analyses were ER, PgR, PS2, age, menopausal status, tumour size, nodal status, disease-free interval between primary surgery and time of relapse, adjuvant chemotherapy and site of metastasis. In the case of multiple sites the lowest score (worst prognosis) was taken. PFS and PRS curves were computed with the method of Kaplan and Meier. The log-rank test was applied to test for differences between two groups in PFS or PRS. The Cox model was used to test for trend. All analyses were restricted to the first 2 years or follow-up because of the low number of patients in the tail of the PFS curves after 2 years.

### Results

### *Patient characteristics*

The characteristics of the patients are listed in Table I. The majority of the patients were post-menopausal, had larger tumours and were node positive at time of primary surgery. The relatively large number of patients with node-positive disease and large (T3,4) tumours, as compared with the general population of patients with primary breast cancer, is probably due to the selection of only patients who developed metastatic disease and to the association of tumour size and nodal status with risk for metastasis. Fourteen per cent of the patients already had metastatic disease at the time of their original diagnosis (disease-free interval of 0). Seventeen per cent of the patients had prior adjuvant chemotherapy. About one-third of the patients who received first-line tamoxifen therapy had primary tumours with ER and/or PgR levels below 10 fmol mg$^{-1}$ protein.

### *Response to treatment*

Of the 230 patients, 41 (18%) showed an objective response (CR, $n = 9$; PR, $n = 32$), 66 (29%) showed a stable disease (SD) of >6 months, 16 (7%) a SD of <6 months, while 107

**Table I** Patient characteristics

| *Patient group* | *Number of patients*[a] | *%* |
|---|---|---|
| All patients | 230 | 100 |
| Menopausal status | | |
| Premenopausal | 45 | 20 |
| Post-menopausal | 185 | 80 |
| Age (years) | | |
| <65 | 126 | 55 |
| ⩾65 | 104 | 45 |
| Nodal status | | |
| N0 | 55 | 26 |
| N1-3 | 55 | 26 |
| N>3 | 105 | 49 |
| Tumour size (cm) | | |
| T1 (⩽2) | 47 | 22 |
| T2 (2-5) | 105 | 50 |
| T3 (>5) | 31 | 15 |
| T4 | 29 | 14 |
| ER level (fmol mg$^{-1}$ protein) | | |
| <10 | 40 | 17 |
| ⩾10 | 190 | 83 |
| PgR level (fmol mg$^{-1}$ protein) | | |
| <10 | 77 | 34 |
| ⩾10 | 152 | 66 |
| Disease main site | | |
| Visceral | 89 | 39 |
| Bone | 102 | 44 |
| Soft tissue | 39 | 17 |
| Disease-free interval | | |
| 0 | 33 | 14 |
| <1 year | 60 | 26 |
| 1-2 years | 77 | 33 |
| >2 years | 60 | 26 |
| Prior adjuvant therapy | | |
| Yes | 38 | 17 |
| No | 192 | 83 |

[a]Owing to missing information numbers do not always add up to 230.

patients showed progressive disease (PD). Median time to progression of patients with a CR was 85.1 months. The rate of progression of patients with a SD of >6 months was not different from that of patients with a PR (median PFS, 12.6 and 13.8 months respectively), whereas patients with a SD <6 months by definition experienced a disease progression between 3 and 6 months (median 5.0 months). Median PFS of patients with PD was 2.5 months.

The median post-relapse survival time (PRS) from start of tamoxifen therapy was 102.9 months for patients with CR, 29.5 months for patients with a PR and 32.2 months for patients with SD >6 months, while median PRS was 9.8 months for patients showing PD and 17.5 months for patients with a SD <6 months. Similar to the criteria used by Ravdin *et al.* (1992), in the present study we have defined response as CR + PR + SD >6 months. Figure 1 shows, for patients with CR, PR, SD >6 months, SD <6 months and PD, the Kaplan–Meier curves for 2-year PRS.

*Levels of PS2 and associations with ER, PgR, and patient characteristics*

The median PS2 level was 7.2 ng mg$^{-1}$ protein (range 0–599 ng mg$^{-1}$ protein; mean ± s.d., 37 ± 74 ng mg$^{-1}$ protein). The level of PS2 was positively correlated with those of ER ($R_s = 0.19$, $P<0.01$) and of PgR ($R_s = 0.23$, $P<0.001$). PS2 was not correlated with menopausal status or age of the patient or with disease-free interval (DFI). PS2 levels were not different in the primary tumours of patients who had had prior adjuvant chemotherapy as compared with those who had had no adjuvant treatment. The disease site was correlated with tumour levels of PS2 ($P<0.02$); primary tumours which later metastasised to the bone had higher PS2 levels (median 12.1 ng mg$^{-1}$ protein) than those that metastasised to visceral sites or to soft tissues (4.3 and 3.2 ng PS2 mg$^{-1}$ protein respectively).

Tumours of patients responding to tamoxifen as compared with non-responding patients had 3.0-fold higher median levels of ER ($P<0.001$), 2.9-fold higher levels of PgR ($P<0.001$), and 2.2-fold higher median levels of PS2 ($P = 0.09$).

*ER, PgR and PS2, and response to tamoxifen therapy*

For tumours with ER or PgR levels equal or above the widely used threshold value of 10 fmol mg$^{-1}$ protein, logistic regression analysis for trend of log transforms of ER and PgR values showed that there was still a significant association of steroid receptor levels ($P = 0.002$ and $P = 0.005$, respectively) with the rate of response (CR + PR + SD >6 months). Therefore it was investigated whether an additional cut-off value for ER and/or PgR could be considered. Based

**Figure 1** Actuarial post-relapse survival for patients with different types of response to tamoxifen therapy CR, complete responders (. . . ); PR, partial responders (——); SD >6 months, stable disease for more than 6 months (---); SD <6 months, stable disease for less than 6 months (-----); PD, progressive disease (---). Numbers between parentheses represent failures/total number of patients in each group.

on isotonic regression analysis with the length of PFS as end point, 75 fmol mg$^{-1}$ protein was chosen as cut-off point for both ER and PgR in addition to 10 fmol mg$^{-1}$ protein. Table II shows the response rates to tamoxifen based on tumour ER and PgR levels <10 fmol mg$^{-1}$ protein, with levels between 10 and 75 fmol mg$^{-1}$ protein, and with levels ⩾75 fmol mg$^{-1}$ protein. For ER the response rate increased from 25% to 63%, and for PgR from 38% to 62%. From a clinical point of view, relevant subgroups could be those with one or both receptors <10 fmol mg$^{-1}$ protein (defined as ER/PgR low), those with both ⩾75 fmol mg$^{-1}$ protein (ER/PgR high), and those with both ⩾10 but not both ⩾75 fmol mg$^{-1}$ protein (ER/PgR intermediate). The response rates increased from 34% for patients with ER/PgR low tumours, via 45% for ER/PgR intermediate tumours, to 66% for patients with ER/PgR high tumours ($P<0.001$, Table II).

In logistic regression analysis for trend there was no significant association between the level of PS2 and the rate of response. A search for an optimised cut-off point for PS2 was considered unjustified. Tumours were therefore arbitrarily divided in three groups. The group with the lowest PS2 levels was defined as those with levels ⩽2 ng mg$^{-1}$ protein, the previously defined cut-off point in analysis for relapse-free survival in primary breast cancer (Foekens *et al.*, 1993). The other groups were defined as those having levels ⩾10 ng mg$^{-1}$ protein, which is the median PS2 level of the ER/PgR intermediate and high tumours (as compared with 1.2 ng mg$^{-1}$ protein of the ER/PgR low group), and a group with PS2 levels in between. The highest response rate (53%) was observed for patients with PS2 levels ⩾10 ng mg$^{-1}$ protein, although this response rate did not significantly differ

**Table II** Response rates to tamoxifen correlated with clinical and cytosolic variables

| *Patient subgroup* | *Number of patients* | *Per cent responding* | P-*value* |
|---|---|---|---|
| All patients | 230 | 47[a] | |
| Menopausal status | | | |
| Premenopausal | 45 | 31 | <0.05 |
| Post-menopausal | 185 | 50 | |
| Age (years) | | | |
| <65 | 126 | 42 | 0.14 |
| ⩾65 | 104 | 52 | |
| ER level (fmol mg$^{-1}$ protein) | | | |
| <10 | 40 | 25 | <0.001 |
| 10–75 | 75 | 32 | |
| ⩾75 | 115 | 63 | |
| PgR level (fmol mg$^{-1}$ protein) | | | |
| <10 | 77 | 38 | <0.005 |
| 10–75 | 76 | 39 | |
| ⩾75 | 76 | 62 | |
| ER/PgR level[b] | | | |
| Low | 87 | 34 | <0.001 |
| Intermediate | 83 | 45 | |
| High | 59 | 66 | |
| PS2 level (ng mg$^{-1}$ protein) | | | |
| ⩽2 | 93 | 43 | 0.20 |
| >2–10 | 37 | 38 | |
| ⩾10 | 100 | 53 | |
| Disease main site | | | |
| Visceral | 89 | 38 | <0.05 |
| Bone | 102 | 48 | |
| Soft tissue | 39 | 62 | |
| Disease-free interval | | | |
| 0 | 33 | 39 | <0.05 |
| <1 year | 60 | 35 | |
| 1–2 years | 77 | 52 | |
| >2 years | 60 | 55 | |
| Prior adjuvant chemotherapy | | | |
| Yes | 38 | 26 | <0.01 |
| No | 192 | 51 | |

[a]Of the 107 responding patients, nine had a CR, 32 a PR and 66 a SD >6 months. [b]Low, one or both <10 fmol mg$^{-1}$ protein; intermediate, both ⩾10 fmol mg$^{-1}$ protein, but not both ⩾75 fmol mg$^{-1}$ protein; high, both ⩾75 fmol mg$^{-1}$ protein.

from those observed for the other two groups with lower PS2 levels (Table II).

Table II furthermore shows that, in univariate analysis, post-menopausal patients experienced a more favourable response rate to tamoxifen treatment than premenopausal patients, while age did not contribute any further, and that soft-tissue and bone metastases showed a higher response rate than visceral metastases. A positive trend was observed regarding disease-free interval and response to tamoxifen therapy. Patients who received prior adjuvant chemotherapy showed a 2-fold lower response rate ($P<0.01$) to tamoxifen therapy of metastatic disease than patients who received no prior adjuvant treatment (Table II). Moreover, the length of PFS and PRS following tamoxifen treatment turned out to be significantly shorter among patients who had received prior adjuvant chemotherapy ($P<0.002$ and $P<0.02$ respectively; Figure 2).

**Figure 2** Actuarial progression-free (top) and post-relapse survival (bottom) for patients with (thin lines) and without (bold lines) prior adjuvant chemotherapy. Numbers between parentheses represent failures/total number of patients in each group.

*ER, PgR, and PS2 and association with PFS and PRS*

The Kaplan–Meier curves for PFS and PRS based on the combined ER/PgR status (left panels) and PS2 status (right panels) are shown in Figure 3. High levels of ER and PgR were associated with a longer PFS and PRS, whereas low or intermediate ER/PgR levels were associated with a more rapid disease progression and death. The PFS curves converged after 2 years, whereas the PRS curves converged only after approximately 4 years (not shown). High levels of PS2 ($\geqslant 10\ \mathrm{ng\ mg^{-1}}$ protein) were also associated with an increased PFS and PRS, although these associations were weaker and in analysis of both PFS and PRS the curves converged after 2 years (Figure 3).

In the relatively large subgroup of 83 patients (= 36%) defined as ER/PgR intermediate, which showed an approximately equally short PFS and PRS following tamoxifen therapy as compared with those defined as ER/PgR low (Figure 3), a high level of PS2 was significantly associated with a prolonged PFS ($P<0.01$) and PRS ($P<0.05$). In this subgroup of tumours with ER/PgR intermediate levels, a high level of PS2 was able to identify those patients with a similar favourable PFS and PRS as those in the ER/PgR high group. In addition, a low level of PS2 could identify patients with a comparable short PFS and PRS as those in the ER/PgR low group (Figure 4 as compared with Figure 3, left panels). In the subgroups defined as ER/PgR low and ER/PgR high, the Kaplan–Meier curves for PFS and PRS stratified by PS2 were superimposable (not shown).

**Figure 3** Actuarial progression-free (top) and post-relapse survival (bottom) stratified by combined ER/PgR status (left) and PS2 status (right). ER/PgR high (bold lines), both $\geqslant 75\ \mathrm{fmol\ mg^{-1}}$ protein; ER/PgR-interim. (dotted lines), both $\geqslant 10\ \mathrm{fmol\ mg^{-1}}$, but not both $\geqslant 75\ \mathrm{fmol\ mg^{-1}}$ protein; ER/PgR low (thin lines), one or both $<10\ \mathrm{fmol\ mg^{-1}}$ protein. PS2 high (bold lines), $\geqslant 10\ \mathrm{ng\ mg^{-1}}$ protein; PS2 low (thin lines), $<10\ \mathrm{ng\ mg^{-1}}$ protein. Numbers between parentheses represent failures/total number of patients in each group.

*Cox multivariate regression analysis for PFS and PRS*

In Cox multivariate regression analyses for PFS and PRS, ER, PgR and PS2 were included together with age and menopausal status, site of metastasis, DFI, size of the primary tumour, nodal status at surgery of the primary tumour and prior adjuvant chemotherapy. In Table III the results of the final Cox multivariate regression analyses for both PFS and PRS are listed. Prior adjuvant chemotherapy was significantly associated with both an early tumour progression (relative hazard rate, RHR 2.29) and an early death (RHR 3.01) after start of tamoxifen therapy for advanced disease. Similarly, visceral metastases were associated with a short PFS (RHR 1.48) and PRS (RHR 2.55). A DFI of more than 1 year was associated with a longer PFS (RHR 0.72), whereas younger patients (<65 years) experienced an earlier death (RHR 1.68). High levels of ER and PgR were associated with a longer PFS (RHR 0.48) and PRS (RHR 0.42). When analysed as a dichotomised or as a continuous variable, PS2 did not contribute significantly to the multivariate models for PFS and PRS for all patients (data not shown).

Separate Cox multivariate analyses for PFS and PRS were performed in the subgroup of 83 tumours defined as ER/PgR intermediate. PS2 was entered as a dichotomised variable ($\geqslant 10$ *vs* $<10\ \mathrm{ng\ mg^{-1}}$ protein). After correction for all other factors as entered in the models presented in Table III, in this subgroup of ER/PgR intermediate tumours, a high level of PS2 was associated with a shorter PFS (RHR 0.51, $P<0.01$) and a shorter PRS (RHR 0.63), the latter not statistically significant ($P = 0.13$).

**Figure 4** Actuarial progression-free (top) and post-relapse survival (bottom) for patients with intermediate ER/PgR tumour levels stratified by PS2. PS2 high (bold lines), $\geqslant 10\ \mathrm{ng\ mg^{-1}}$ protein; PS2 low (thin lines), $<10\ \mathrm{ng\ mg^{-1}}$ protein. Numbers between parentheses represent failures/total number of patients in each group.

### Discussion

For refinement of the selection of therapy, it would be beneficial to have other factors available, additional to ER and PgR, which could more reliably identify those patients likely to respond or fail to respond to endocrine therapy of advanced breast cancer. One candidate factor could prove to be PS2, a protein which is secreted by breast cancer cells *in vitro* upon oestrogen stimulation (Masiakowski *et al.*, 1982; Nunez *et al.*, 1987). Indeed, in a few pilot studies involving up to 72 patients, PS2 transcripts (Henry *et al.*, 1989; Skilton *et al.*, 1989) and immunohistochemically assessed PS2 status (Henry *et al.*, 1991; Schwartz *et al.*, 1991) were reported to be predictive for endocrine responsiveness of advanced breast cancer. However, no consensus exists regarding immunohistochemically assessed PS2 status (Luqmani *et al.*, 1993*a*). In only two studies described so far, PS2 status has been studied in relation to duration of post-relapse survival and/or duration of response, but no association was found (Henry *et al.*, 1989; Schwartz *et al.*, 1991).

Higher levels of PS2, measured by immunoassays in breast tumour cytosols, have shown to be associated with an increased length of relapse-free and overall survival in primary breast cancer (Foekens *et al.*, 1990, 1993; Predine *et al.*, 1992; Gion *et al.*, 1993) and a more favourable response to adjuvant hormonal therapy (Predine *et al.*, 1992). However, no information is currently available in the literature regarding cytosolic PS2 levels and response to endocrine therapy for advanced disease. Irrespective of the technique used to assess PS2, as in the present study, the expression of PS2 in human primary breast tumours has shown to be correlated with that of ER or PgR (Rio *et al.*, 1987; Henry *et al.*, 1989, 1991; Skilton *et al.*, 1989; Foekens *et al.*, 1990, 1993; Schwartz *et al.*, 1991; Cappelletti *et al.*, 1992; Koerner *et al.*, 1992; Predine *et al.*, 1992; Thor *et al.*, 1992; Gion *et al.*, 1993; Luqmani *et al.*, 1993*b*; Thompson *et al.*, 1993).

In the present study, PS2 was quantitatively assessed in cytosols of a relatively large number of 230 tumours. The log of PS2 showed a positive association with the probability of response and PFS, but these associations were not very strong and not statistically significant. To enable visualisation of the effects of PS2 on progression-free survival (PFS) and post-relapse survival (PRS), and for reasons of more convenient data analyses, we have chosen to dichotomise PS2 values at $10\ \mathrm{ng\ mg^{-1}}$ protein, which was the median value in tumours not belonging to the subgroup with low ER and/or PgR levels. Patients with high tumour levels of PS2 experienced a prolonged PFS and PRS, although the associations were weak and of borderline or no statistical significance (Figure 3). From the results listed in Table II and shown in Figure 3, it can be concluded that tumours with intermediate ER/PgR levels respond approximately equally poorly to

**Table III** Cox multivariate analysis for progression-free and post-relapse survival[a]

| | *Progression-free survival* | | *Post-relapse survival* | |
|---|---|---|---|---|
| | *RHR (95% CL)*[b] | *P-value* | *RHR (95% CL)*[b] | *P-value* |
| Age[c] | 1.05 (0.75–1.45) | 0.76 | 1.68 (1.12–2.51) | 0.01 |
| Disease-free interval[d] | 0.72 (0.53–0.98) | 0.04 | 0.71 (0.48–1.03) | 0.07 |
| Visceral metastasis[e] | 1.48 (1.10–2.00) | <0.01 | 2.55 (1.78–3.66) | <0.001 |
| ER/PgR[f] | 0.48 (0.34–0.68) | <0.001 | 0.42 (0.26–0.66) | <0.001 |
| Adjuvant therapy[g] | 2.29 (1.45–3.42) | <0.001 | 3.01 (1.74–5.21) | <0.001 |

[a]The models included 229 patients. Tumour size, nodal status and menopausal status did not significantly contribute to the models. [b]RHR (95% CL): relative hazard rate (95% confidence limits). [c]Age: <65 years versus $\geqslant 65$ years. [d]Disease-free interval: $\geqslant 1$ year versus <1 year. [e]Visceral metastasis: yes versus no. [f]ER/PgR-high (both $\geqslant 75\ \mathrm{fmol\ mg^{-1}}$ protein versus ER/PgR intermediate and ER/PgR low. [g]Adjuvant therapy: prior adjuvant chemotherapy, yes versus no.

tamoxifen therapy as those having low ER/PgR levels. Therefore, it is questionable whether patients with this ER/PgR intermediate phenotype, a relatively large group of tumours (36% of the total number of patients), should be treated with tamoxifen based on ER and PgR values alone. Interestingly, in the exploratory analyses shown in Figure 4, the predictive effect of PS2 in analyses for PFS ($P<0.01$) and PRS ($P<0.05$) was exclusively present in the subgroup of tumours with intermediate ER and PgR levels (also in multivariate analysis for PFS), and not in the subgroups with low or high ER and PgR values. In this subgroup, PS2 identified patients with a good and poor prognosis, such that tumours with low PS2 levels behaved similarly to those with low ER/PgR levels and tumours with high PS2 levels performed comparably well as those having both high ER and PgR levels.

In the present study PS2 status as determined by quantitative immunoassay in cytosolic extracts had less predictive power than ER or PgR status. These data are in agreement with those of Luqmani et al. (1993a), who used immunohistochemically assessed ER, PgR and PS2 in a series of 70 patients. On the other hand, Henry et al. (1991) in a study involving 35 patients, and Schwartz et al. (1991) in a study involving 72 patients, found immunohistochemically assessed PS2 positivity to be a stronger predictive factor for response to endocrine therapy than ER. Moreover, PS2 mRNA expression was found to be a stronger predictive factor for response to tamoxifen therapy than ER mRNA expression in a study involving 21 patients (Henry et al., 1989), whereas Skilton et al. (1989) in a series of 21 patients reported an equal response rate when analysing PS2 mRNA expression and ER expression determined by ligand-binding assay or by immunohistochemistry. The low number of patients in the various studies and the different techniques used to assess PS2 status most likely caused the discrepancies between the results obtained.

ER and also PgR levels were found to be significantly predictive for response (Table II) duration of response (Figure 3) and length of post-relapse survival (Figure 3) after the start of tamoxifen therapy for advanced disease. In addition, we found that the higher the levels of ER or PgR, the higher the likelihood of a response, and consequently longer PFS and PRS. Cox regression models of PFS and PRS showed that after correction for the effect of other prognostic indicators, ER and PgR remained as significant independent variables. Similar results were recently reported by Ravdin et al. (1992) describing a prospective evaluation of PgR in ER-positive breast cancer patients treated with tamoxifen.

The overall response rate (CR, PR and SD $>6$ months) in this study, involving also 40 (17%) patients with tumours containing ER levels $<10\,\mathrm{fmol\,mg^{-1}}$, was 47%. This is similar to the overall response rate of 54% reported by Ravdin et al. (1992) using the same response criteria in a study involving only patients with ER levels more than $3\,\mathrm{fmol\,mg^{-1}}$ protein. Similarly, the lower response rate in premenopausal patients as compared with post-menopausal patients in our study (30% vs 50%) was similar to that reported by Ravdin et al. (1992) (24% vs 57%). This low response rate in premenopausal patients may be due to the lower percentage of tumours with ER levels $\geqslant 75\,\mathrm{fmol\,mg^{-1}}$ protein as compared with tumours of post-menopausal patients (27% vs 56%, $P<0.001$) in the present study. As a result of the stronger contribution of ER in the multivariate models for PFS and PRS, menopausal status was not an independent variable.

Interestingly, patients with a DFI of less than 1 year showed a lower probability and shorter duration of response, a finding also described by Ravdin et al. (1992). The reason for this is currently unclear but might have a biological basis, i.e. that rapidly relapsing tumours have a more aggressive phenotype and as result are subsequently prone to treatment failure once metastasised. In contrast to the study of Ravdin et al. (1992), in the present study the site of disease, particularly visceral metastasis, was a significant predictor of lower probability of response to therapy and a shorter PFS and PRS. This was not because in our study the primary tumours of patients who developed visceral metastases had lower ER or PgR levels which could explain the lower response rates. Moreover, the association of visceral metastasis with PFS and PRS was also present in the multivariate models presented in Table III.

Of 38 patients who had received adjuvant chemotherapy, only 26% responded, as compared with 51% of the patients who did not receive adjuvant treatment (Table II). Consequently, patients who had received adjuvant treatment experienced a significantly shorter PFS and PRS (Figure 2) in multivariate analyses also (Table III). These observations were not made by Ravdin et al. (1992), but were observed by others (for review see, Rubens et al., 1994), and a 2-fold difference in response was also obtained by the Guy's Hospital, as reviewed by Rubens (1993). Moreover, a shorter PFS and PRS were observed, although the relationship between prior adjuvant chemotherapy and shorter PRS was not statistically significant since the curves converged with time (Rubens, 1993). A similar pattern of the survival curves with time has also been reported by de Takats et al. (1993). The observation that patients who received adjuvant treatment respond worse to subsequent tamoxifen therapy on relapse may suggest that prior chemotherapy caused a selection of specific tumour cells with an altered hormone receptor phenotype in the occult tumours, possibly by chemical castration. Nevertheless, for the overall group of primary breast cancer patients the results of the meta-analysis (Early Breast Cancer Trialists' Collaborative Group, 1992) indicate that the positive benefit on overall survival is not overriden by shortened post-relapse survival in patients with relapse.

From the current study we conclude that, as compared with PS2, cytosolic ER and PgR are stronger markers to predict response, duration of response and length of post-relapse survival on tamoxifen therapy for advanced disease. In addition to ER and PgR, PS2 status may, however, be helpful in defining those patients with intermediate ER and PgR levels who are likely to benefit from tamoxifen therapy. Further prospective studies are necessary before coming to firm conclusions on the usefulness of PS2 as an additional marker to predict the hormonal responsiveness of advanced breast cancer.

We wish to thank the expert technical assistance of Mrs E. Binnendijk-Noordegraaf, Mrs E.M.J. Stuurman-Smeets and Mr H.A. Peters, and we thank Drs Y.W.C.M. de Koning and M.J. Hooning for their assistance with the collection of the clinical data.

This work was supported by a grant from the Dutch Cancer Society, Project DDHK 92-04.

### References

CAPPELLETTI, V., CORADINI, D., SCANZIANI, E., BENINI, E., SILVESTRINI, R. & DI FRONZO, G. (1992). Prognostic relevance of pS2 status in association with steroid receptor status and proliferative activity in node-negative breast cancer. *Eur. J. Cancer*, **28A**, 1315-1318.

DE TAKATS, P.G., DUNN, J.A., KERR, D.J. & MORRISON, J.M. (1993). Impact of adjuvant chemotherapy in breast cancer on response to tamoxifen on relapse. *Cancer Treat. Rev.*, **19** (Suppl. B), 11-19.

EARLY BREAST CANCER TRIALISTS' COLLABORATIVE GROUP (1992). Systemic treatment of early breast cancer by hormonal, cytotoxic or immune therapy. *Lancet*, **339**, 1-15, 71-85.

EORTC BREAST CANCER COOPERATIVE GROUP (1980). Revision of the standards for the assessment of hormone receptors in human breast cancer. *Eur. J. Cancer*, **16**, 1513-1515.

FOEKENS, J.A., PORTENGEN, H., VAN PUTTEN, W.L.J., PETERS, H.A., KRIJNEN, H.L.J.M., ALEXIEVA-FIGUSCH, J. & KLIJN, J.G.M. (1989). Prognostic value of estrogen and progesterone receptors measured by enzyme immunoassays in human breast tumor cytosols. *Cancer Res.*, **49**, 5823-5828.

FOEKENS, J.A., RIO, M.-C., SEGUIN, P., VAN PUTTEN, W.L.J., FAUQUE, J., NAP, M., KLIJN, J.G.M. & CHAMBON, P. (1990). Prediction of relapse and survival in breast cancer patients by pS2 protein status. *Cancer Res.*, **50**, 3832-3837.

FOEKENS, J.A., VAN PUTTEN, W.L.J., PORTENGEN, H., DE KONING, Y.W.C.M., THIRION, B., ALEXIEVA-FIGUSCH, J. & KLIJN, J.G.M. (1993). Prognostic value of PS2 and cathepsin D in 710 human primary breast tumors: multivariate analysis. *J. Clin. Oncol.*, **11**, 899-908.

FUQUA, S.A.W., FITZGERALD, S.D., ALLRED, D.C., ELLEDGE, R.M., NAWAZ, Z., MCDONNELL, D.P., O'MALLEY, B.W., GREENE, G.L. & MCGUIRE, W.L. (1992). Inhibition of estrogen receptor action by a naturally occurring variant in human breast tumors. *Cancer Res.*, **52**, 483-486.

FUQUA, S.A.W., CHAMNESS, G.C. & MCGUIRE, W.L. (1993*a*). Estrogen receptor mutations in breast cancer. *J. Cell. Biochem.*, **51**, 135-139.

FUQUA, S.A.W., ALLRED, D.C., ELLEDGE, R.M., KRIEG, S.L., BENEDIX, M.G., NAWAZ, Z., O'MALLEY, B.W., GREENE, G.L. & MCGUIRE, W.L. (1993*b*). The ER-positive/PgR-negative breast cancer phenotype is not associated with mutations within the DNA binding domain. *Breast Cancer Res. Treat.*, **26**, 191-202.

GASPARINI, G., POZZA, F. & HARRIS, A.L. (1993). Evaluating the potential usefulness of new prognostic and predictive indicators in node-negative breast cancer patients. *J. Natl Cancer Inst.*, **85**, 1206-1219.

GION, M., MIONE, R., PAPPAGALLO, G.L., GATTI, C., NASCIMBEN, O., BARI, M., LEON, A.E., VINANTE, O. & BRUSCAGNIN, G. (1993). PS2 in breast cancer - alternative or complementary tool to steroid receptor status? Evaluation of 446 cases. *Br. J. Cancer*, **68**, 374-379.

HENRY, J.A., NICHOLSON, S., HENNESSY, C., LENNARD, T.W.J., MAY, F.E.B. & WESTLEY, B.R. (1989). Expression of the oestrogen regulated pNR-2 mRNA in human breast cancer: relation to oestrogen receptor regulated pNR-2 mRNA in human breast cancer: relation to oestrogen receptor mRNA levels and response to tamoxifen therapy. *Br. J. Cancer*, **61**, 32-38.

HENRY, J.A., PIGGOTT, N.H., MALLICK, U.K., NICHOLSON, S., FARNDON, J.R., WESTLEY, B.R. & MAY, F.E.B. (1991). pNR-2/PS2 immuno-histochemical staining in breast cancer: correlation with prognostic factors and endocrine response. *Br. J. Cancer*, **63**, 615-622.

HORWITZ, K.B. (1993). Mechanisms of hormone resistance in breast cancer. *Breast Cancer Res. Treat.*, **26**, 119-130.

HORWITZ, K.B., MCGUIRE, W.L., PEARSON, O.A. & SEGALOFF, A. (1975). Predicting response to endocrine therapy in human breast cancer: a hypothesis. *Science*, **189**, 726-727.

HORWITZ, K.B., WEI, L.L., SEDLACEK, S.M. & D'ARVILLE, C.N. (1985). Progestin action and progesterone receptor structure in human breast cancer: a review. *Recent Prog. Horm. Res.*, **41**, 249-316.

KLIJN, J.G.M. & FOEKENS, J.A. (1990). Prognostic factors in breast cancer. In *Endocrine Therapy of Breast Cancer IV*. Goldhirsch, A. (ed.), Monographs of the European School of Oncology, pp. 17-25, Springer: Berlin.

KLIJN, J.G.M., BERNS, E.M.J.J., VAN PUTTEN, W.L.J., DE KONING, Y.W.C.M., ALEXIEVA-FIGUSCH, J., BONTENBAL, M. & FOEKENS, J.A. (1992). The prognostic value of oncogene amplification and of tumoral secretory proteins with respect to response to endocrine and chemotherapy in metastatic breast cancer (abstract). *Proc. Am. Soc. Clin. Oncol.*, **11**, 53-57.

KLIJN, J.G.M., BERNS, E.M.J.J., BONTENBAL, M. & FOEKENS, J.A. (1993). Cell biological factors associated with the response of breast cancer to systemic treatment. *Cancer Treat. Rev.*, **19** (Suppl. B), 45-63.

KOERNER, F.C., GOLDBERG, D.E., EDGERTON, S.M. & SCHWARTZ, L.H. (1992). pS2 protein and steroid hormone receptors in invasive breast carcinomas. *Int. J. Cancer*, **52**, 183-188.

LUQMANI, Y.A., RICKETTS, D., RYALL, G., TURNBULL, L., LAW, M. & COOMBES, R.C. (1993*a*). Prediction of response to endocrine therapy in breast cancer using immunohistochemical assays for PS2, oestrogen receptor and progesterone receptor. *Int. J. Cancer*, **54**, 619-623.

LUQMANI, Y.A., CAMPBELL, T., SOOMRO, S., SHOUSHA, S., RIO, M.C. & COOMBES, R.C. (1993*b*). Immunohistochemical localisation of pS2 protein in ductal carcinoma in situ and benign lesions of the breast. *Br. J. Cancer*, **67**, 749-753.

MASIAKOWSKI, P., BREATHNACH, R., BLOCH, J., GANNON, F., KRUST, A. & CHAMBON, P. (1982). Cloning of cDNA sequences of hormone-regulated genes from the MCF-7 human breast cancer cell line. *Nucleic. Acids Res.*, **10**, 7895-7903.

MCGUIRE, W.L. & CLARK, G.M. (1992). Prognostic factors and treatment decisions in axillary node-negative breast cancer patients. *N. Engl. J. Med.*, **326**, 1756-1761.

NUNEZ, A.M., JACOLEW, S., BRIAND, J.P., GAIRE, M., KRUST, A., RIO, M.-C. & CHAMBON, P. (1987). Characterization of the estrogen-induced pS2 protein secreted by the human breast cancer cell line MCF-7. *Endocrinology*, **121**, 1759-1765.

OSBORNE, C.K., YOCHMOWITZ, M.G., KNIGHT, III, W.A. & MCGUIRE, W.L. (1980). The value of estrogen and progesterone receptors in the treatment of breast cancer. *Cancer*, **46**, 2884-2888.

PREDINE, J., SPYRATOS, F., PRUD'HOMME, J.F., ANDRIEU, C., HACENE, K., BRUNET, M., PALLUD, C. & MILGROM, E. (1992). Enzyme-linked immunosorbent assay of pS2 in breast cancers, benign tumours, and normal tissues. *Cancer*, **69**, 2116-2123.

RAVDIN, P.M., GREEN, S., MELINK DORR, T., MCGUIRE, W.L., FABIAN, C., PUGH, R.P., CARTER, R.D., RIVKIN, S.E., BORST, J.R., BELT, R.J., METCH, B. & OSBORNE, C.K. (1992). Prognostic significance of progesterone receptor levels in estrogen receptor-positive patients with metastatic breast cancer treated with tamoxifen: results of a prospective Southwest Oncology Group study. *J. Clin. Oncol.*, **10**, 1284-1291.

RIO, M.-C., BELLOCQ, J.P., GAIRARD, B., RASMUSSEN, B., KRUST, A., KOEHL, C., CALDEROLI, H., SCHIFF, V., RENAUD, R. & CHAMBON, P. (1987). Specific expression of the pS2 gene in subclasses of breast cancers in comparison with the expression of the estrogen and progesterone receptors and the oncogene erbB2. *Proc. Natl Acad. Sci. USA*, **84**, 9243-9247.

RUBENS, R.D. (1993). Effect of adjuvant systemic therapy on response to treatment after relapse. *Cancer Treat. Rev.*, **19** (Suppl. B), 3-10.

RUBENS, R.D., BAJETTA, E., BONNETERRE, J., KLIJN, J.G.M., LONNING, P.E. & PARIDAENS, R. (1994). Treatment of relapse of breast cancer after adjuvant systemic therapy: review and guidelines for future research. *Eur. J. Cancer*, **30A**, 106-111.

SCHWARTZ, L.H., KOERNER, F.C., EDGERTON, S.M., SAWICKA, J.M., RIO, M.-C., BELLOCQ, J.P., CHAMBON, P. & THOR, A.D. (1991). pS2 expression and response to hormonal therapy in patients with advanced breast cancer. *Cancer Res.*, **51**, 624-628.

SCOTT, G.K., KUSHNER, P., VIGNE, J.-L. & BENZ, C.C. (1991). Truncated forms of DNA-binding estrogen receptors in human breast cancer. *J. Clin. Invest.*, **88**, 700-706.

SKILTON, R.A., LUQMANI, Y.A., MCCLELLAND, R.A. & COOMBES, R.C. (1989). Characterization of a messenger RNA selectively expressed in human breast cancer. *Br. J. Cancer*, **60**, 168-175.

SLUYSER, M. & MESTER, J. (1985). Oncogenes homologous to steroid receptors? *Nature*, **315**, 546.

THOMPSON, A.M., HAWKINS, R.A., ELTON, R.A., STEEL, C.M., CHETTY, U. & CARTER, D.C. (1993). pS2 is an independent factor of good prognosis in primary breast cancer. *Br. J. Cancer*, **68**, 93-96.

THOR, A.D., KOERNER, F.C., EDGERTON, S.M., WOOD, W.C., STRACHER, M.A. & SCHWARTZ, L.H. (1992). pS2 expression in primary breast carcinomas: relationship to clinical and histological features and survival. *Breast Cancer Res. Treat.*, **21**, 111-119.